# Expression levels and DNA methylation profiles of the growth gene *SHOX* in cartilage tissues and chondrocytes

**DOI:** 10.1038/s41598-024-58530-9

**Published:** 2024-04-05

**Authors:** Atsushi Hattori, Atsuhito Seki, Naoto Inaba, Kazuhiko Nakabayashi, Kazue Takeda, Kuniko Tatsusmi, Yasuhiro Naiki, Akie Nakamura, Keisuke Ishiwata, Kenji Matsumoto, Michiyo Nasu, Kohji Okamura, Toshimi Michigami, Yuko Katoh-Fukui, Akihiro Umezawa, Tsutomu Ogata, Masayo Kagami, Maki Fukami

**Affiliations:** 1 Department of Molecular Endocrinology, National Research Institute for Child Health and Development, Tokyo, 157-8535 Japan; 2 Division of Diversity Research, National Research Institute for Child Health and Development, Tokyo, 157-8535 Japan; 3 Department of Orthopaedic Surgery, National Medical Center for Children and Mothers, Tokyo, 157-8535 Japan; 4 Department of Maternal-Fetal Biology, National Research Institute for Child Health and Development, Tokyo, 157-8535 Japan; 5 Department of Allergy and Clinical Immunology, National Research Institute for Child Health and Development, Tokyo, 157-8535 Japan; 6 Center for Regenerative Medicine, National Research Institute for Child Health and Development, Tokyo, 157-8535 Japan; 7 Division of Endocrinology and Metabolism, National Medical Center for Children and Mothers, Tokyo, 157-8535 Japan; 8 Department of Systems BioMedicine, National Research Institute for Child Health and Development, Tokyo, 157-8535 Japan; 9 Department of Bone and Mineral Research, Research Institute, Osaka Women’s and Children’s Hospital, Osaka Prefectural Hospital Organization, Izumi, 594-1101 Japan; 10 Department of Pediatrics, Hamamatsu University School of Medicine, Hamamatsu, 431-3192 Japan; 11 Department of Pediatrics, Hamamatsu Medical Center, Hamamatsu, 432-8580 Japan

**Keywords:** DNA methylation, Gene expression, Genetics research, Epigenetic memory

## Abstract

All attempts to identify male-specific growth genes in humans have failed. This study aimed to clarify why men are taller than women. Microarray-based transcriptome analysis of the cartilage tissues of four adults and chondrocytes of 12 children showed that the median expression levels of *SHOX*, a growth gene in the pseudoautosomal region (PAR), were higher in male samples than in female samples. Male-dominant *SHOX* expression was confirmed by quantitative RT-PCR for 36 cartilage samples. Reduced representation bisulfite sequencing of four cartilage samples revealed sex-biased DNA methylation in the *SHOX*-flanking regions, and pyrosequencing of 22 cartilage samples confirmed male-dominant DNA methylation at the CpG sites in the *SHOX* upstream region and exon 6a. DNA methylation indexes of these regions were positively correlated with *SHOX* expression levels. These results, together with prior findings that PAR genes often exhibit male-dominant expression, imply that the relatively low *SHOX* expression in female cartilage tissues reflects the partial spread of X chromosome inactivation into PAR. Altogether, this study provides the first indication that sex differences in height are ascribed, at least in part, to the sex-dependent epigenetic regulation of *SHOX*. Our findings deserve further validation.

## Introduction

Mean adult height of men is ~ 13 cm greater than that of women^1,2^. This sexual dimorphism is primarily ascribed to the effects of sex hormones and sex chromosomal genes^1^. Considering that individuals with 46,XY gonadal dysgenesis are in general ~ 8 cm taller than those with 46,XX gonadal dysgenesis^1^, sex chromosomal genes likely play a more prominent role in male-dominant growth than sex hormones. To date, however, all attempts to identify a male-specific growth gene have failed. Actually, no major growth gene has been identified on the human sex chromosomes, except for *SHOX* which resides in the short arm pseudoautosomal region (PAR1) of the X and Y chromosomes^3,4^. *SHOX* encodes a regulator of chondrocyte development, and its haploinsufficiency typically causes a height reduction of more than 10 cm^4,5^. *SHOX* was assumed to exert equivalent beneficial effects on the growth of men and women, because all PAR1 genes, including *SHOX*, were believed to escape X chromosome inactivation (XCI) and show biallelic expression in both 46,XX and 46,XY cells^3,6^.

In the present study, we tested a new hypothesis that *SHOX* undergoes sex-dependent epigenetic regulation. This hypothesis is based on the previous findings by Tukiainen et al. that the average transcript levels of PAR1 genes in various female tissues were lower than those in male tissues^7^. Tukiainen et al. proposed that the male-dominant expression of PAR1 genes reflects the partial spread of XCI into PAR1, because (i) transcript levels of these genes from the inactive X chromosome (Xi) accounted for only ~ 80% of those from the active X chromosome (Xa), and (ii) PAR1 genes were not up- or down-regulated on the Y chromosome (Y). However, Tukiainen et al. did not analyze transcript levels in cartilage tissues or chondrocytes. Moreover, the authors did not investigate DNA methylation profiles, even though XCI is known to alter DNA methylation profiles of target genes^8^. For example, Cotton et al. have reported that the average methylation rates of the promoter/enhancer regions of XCI-subject and -escape genes on Xi were 70% and 11% respectively, whereas those of gene bodies were 64% and 75% respectively^8^. In the present study, we analyzed transcript levels and DNA methylation profiles of X chromosomal genes, including *SHOX*, in the cartilage tissues and chondrocytes of men and women.

## Results

### Microarray-based transcriptome analysis of X chromosomal genes

**Figure 1:**
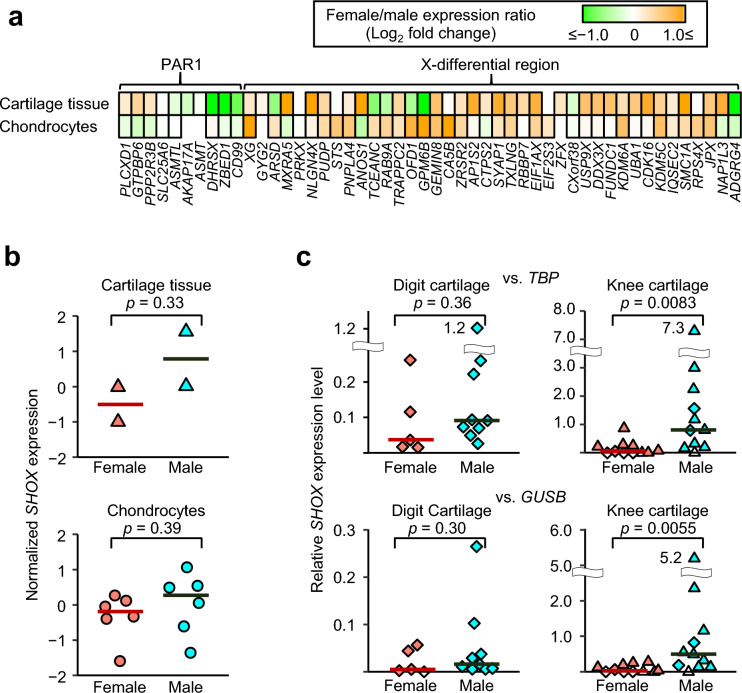
Representative results of mRNA quantification. (**a**) Microarray-based transcriptome analysis of X chromosomal genes. We analyzed cartilage tissues obtained from two women and two men and cultured chondrocytes established from six girls and six boys. Female/male expression ratios of X chromosome inactivation (XCI)-escape genes that passed the filtering criteria are shown. PAR1, the short arm pseudoautosomal region. (**b**) *SHOX* expression levels calculated from transcriptome data. The value was manually calculated by subtracting the median log2 value of *SHOX* expression levels of all samples from the value of each sample. The horizontal bars indicate the median values of the female and male groups. (**c**) RT-qPCR analysis of cartilage tissues. The results of knee cartilage tissues from 22 adolescent/adults and digit cartilage tissues from 14 children are shown. Relative *SHOX* expression levels were analyzed using two internal controls (*TBP* and *GUSB*). The diamond and triangle symbols indicate fresh frozen and RNAlater-treated tissues, respectively. The horizontal bars depict the median values of the female and male groups. Unfilled symbols indicate values lower than the detection limit.

We first examined sex differences in transcript levels of X chromosomal genes. Microarray-based transcriptome analysis was performed using knee cartilage tissues obtained from four adults (two women and two men) and cultured chondrocytes established from the fingers of 12 children (six girls and six boys) (Supplementary Table S1 online). The results showed relatively high expression of PAR1 genes in the male samples, together with the female-dominant expression of known XCI-escape genes in the X-differential region (Fig. 1a). These data are consistent with the previous report by Tukiainen et al.^7^ Since *SHOX* did not pass the filtering criteria due to low expression levels in some samples, we analyzed the data manually. The results showed that the median *SHOX* expression levels were higher in the male samples than those in the female samples albeit not reaching statistical significance (Fig. 1b).

### RT-qPCR analysis of *SHOX*

To validate the results of the microarray-based transcriptome analysis, we performed RT-qPCR analysis of *SHOX* using knee cartilage tissues obtained from 22 adolescent/adults (11 women and 11 men) and digit cartilage tissues obtained from 14 children (five girls and nine boys). Relative *SHOX* expression levels were lower in the digit cartilage tissues than in the knee cartilage tissues. In both groups, the median values of relative *SHOX* expression were higher in the male samples than in the female samples (Fig. 1c). The differences in knee cartilage samples reached statistical significance. No correlation was observed between *SHOX* expression levels and height in five children (Supplementary Fig. S1 online), while height data of other individuals were not available.

### DNA methylation analyses of X chromosomal genes

**Figure 2:**
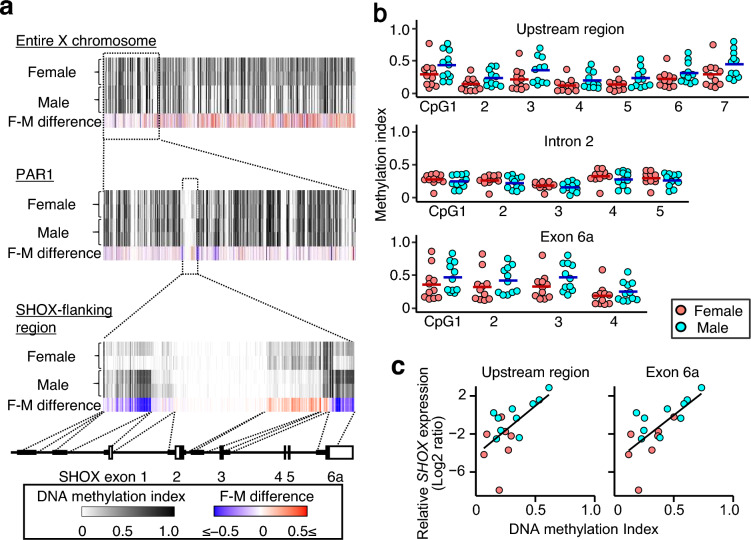
Representative results of DNA methylation analyses. (**a**) Reduced representation bisulfite sequencing (RRBS) of the cartilage tissues obtained from two women and two men. A DNA methylation index is defined as the ratio of 5-methylcytosines to total cytosines. For each CpG site, we calculated the difference in the mean methylation index between the female and male samples (“F-M difference”). The white and black boxes depict non-coding and coding exons of *SHOX*, respectively. PAR1, pseudoautosomal region 1. (**b**) Pyrosequencing of knee cartilage tissues obtained from 11 women and 11 men. Methylation indexes of 16 CpG sites in the *SHOX*-flanking regions (*SHOX*-upstream region, intron 2, and exon 6a) are shown. The horizontal bars describe the mean value of each group. The *P* values regarding the sex difference in DNA methylation are described below. Upstream region: CpG1, 0.1223; CpG2, 0.06164; CpG3, 0.09916; CpG4, 0.1387; CpG5, 0.08258; CpG6, 0.1294; CpG7, 0.07291. Intron 2: CpG1, 0.4622; CpG2, 0.3061; CpG3, 0.314; CpG4, 0.3213; CpG5, 0.3822. Exon 6a: CpG1, 0.2769; CpG2, 0.3137; CpG3, 0.1179; CpG4, 0.3256. (**c**) Correlations between *SHOX* expression levels and DNA methylation indexes at CpG sites in the flanking regions. The X-axis represents the average DNA methylation index at CpG sites in each region: the *SHOX* upstream and exon 6a. The Y-axis represents the log2-transformed relative *SHOX* expression levels against *TBP*. Linear regression lines fitted to the data are shown. The correlation coefficients and *P* values for the relationship between DNA methylation index and *SHOX* expression in each region are listed below. Upstream region: coefficient = 0.61 and *P* value = 0.0090. Exon 6a: coefficient = 0.70 and *P* value = 0.0018.

Next, we analyzed DNA methylation profiles of X chromosomal genes. Reduced representation bisulfite sequencing (RRBS) was performed using four knee cartilage tissues obtained from adults (two women and two men). RRBS is a method to assess DNA methylation profiles of CpG-rich genomic regions^9^. The results showed that most CpG sites in the X-differential region were more methylated in the female samples than in the male samples, whereas such female-dominant DNA methylation was not apparent for CpG sites in PAR1 (Fig. 2a). Of note, we detected sex differences in the methylation indexes of three CpG clusters around *SHOX* (Fig. 2a). Specifically, male-dominant DNA methylation was observed in a 3.2 kb region that is 3.9 kb upstream of *SHOX* (chrX:578,055–581,224; GRCh37/hg19) and a 1.9 kb region in intron 5–exon 6a (chrX:604,185–606,039), whereas female-dominant DNA methylation was detected in a 1.2 kb region in intron 2 (chrX:592,562–593,801) (Fig. 2a).

To validate the results of RRBS, we conducted pyrosequencing of 16 CpG sites in the *SHOX*-flanking regions of 22 knee cartilage tissues. The results showed little sex difference in the DNA methylation of CpG sites in intron 2 (Fig. 2b). In contrast, DNA methylation patterns in the upstream region and exon 6a were consistent with the results of RRBS. The average DNA methylation indexes of CpG sites in these regions were invariably lower in the female samples than in the male samples (Fig. 2b). DNA methylation indexes of CpG sites in the upstream region and exon 6a were positively correlated with *SHOX* expression levels (Fig. 2c).

## Discussion

This study showed male-dominant *SHOX* expression in cartilage tissues and chondrocytes. The sex differences reached statistical significance in knee cartilage tissues (Fig. 1c). The lack of statistical significance in the child cartilage samples appears to reflect the small sample size and low *SHOX* expression levels (Fig. 1b,c). Since *SHOX* is known to facilitate growth in a dosage-sensitive manner^4^, relatively low *SHOX* expression in female cartilage tissues likely contributes to the relatively short stature of women. This speculation is supported by the fact that female patients with *SHOX* haploinsufficiency usually manifest more severe phenotypes than male patients^4,5,10^. Moreover, the similar phenotypes of male patients with *SHOX* defects on X and Y (Supplementary Table S2 online) suggest that in men, *SHOX* is equally expressed from the two sex chromosomes. The lack of *SHOX* orthologs in rodents and several other species^3^ may explain why sex-biased expression of *Shox* has not been reported to date. Notably, we observed relatively large variations in *SHOX* expression levels in both the male and female groups. Such variations may contribute to interindividual height variations within the same sex. However, since the height data of most participants were unavailable, this notion needs to be examined in future studies.

Microarray-based transcriptome analysis of cartilage tissues showed that most PAR1 genes, including *SHOX*, were more strongly expressed in the male samples than in the female samples. These results are consistent with previous data obtained from other tissues^7^, and support the notion that PAR1 genes are subjected to partial XCI. Notably, RRBS showed sex-biased DNA methylation at the *SHOX*-flanking CpG sites, and pyrosequencing confirmed male-dominant DNA methylation in the *SHOX* upstream region and exon 6a. The hypomethylation of exon 6a in the female samples can be regarded as a sign of XCI-subject genes, because previous studies have shown that Xa of women and the single X of men have similar DNA methylation profiles^11^ and that XCI-subject genes on Xi typically exhibit relative hypomethylation in gene bodies^8,12^. Likewise, male-dominant DNA methylation in the upstream region is consistent with the previous findings that intergenic CpG sites on Xa are usually more methylated than those on Xi^8^. Moreover, we found that *SHOX* expression levels were positively correlated with DNA methylation indexes at CpG sites in the upstream region and exon 6. Thus, DNA methylation of these CpG sites may be a part of the epigenetic regulation of *SHOX*. Altogether, our data argue for the partial XCI on *SHOX*. This assumption is supported by the previous findings by Sun et al. that two women with extremely skewed XCI manifested diverse phenotypes despite having the same heterozygous *SHOX* deletion^13^. Given the small number of our subjects, the results of this study need to be validated in future studies. Moreover, it is necessary to clarify allele-specific DNA methylation profiles of *SHOX*-flanking CpG sites in male and female samples.

## Conclusions

This study suggests the male-dominant expression and sex-biased DNA methylation of *SHOX* in cartilage tissues and chondrocytes, which possibly reflects incomplete spread of XCI into PAR1. Such sex-dependent epigenetic regulation of *SHOX* likely contributes to sex differences in adult height. Our findings deserve further validation.

## Methods

### Ethical approval

This study was approved by the Institutional Review Board Committee at the National Center for Child Health and Development (project #1763). Written informed consent was obtained from the sample donors and/or their parents. All methods were performed in accordance with the relevant guidelines and regulations.

### The target gene *SHOX*

This study primarily focused on *SHOX*a, the major transcript of *SHOX* (NM_000451.3). *SHOX*a consists of exons 1–6a and resides at chrX:585,079–607,558 and chrY:535,079–557,558 (GRCh37/hg19). Although previous studies have identified several splice variants of *SHOX*, all of these variants except for *SHOX*a are of unknown clinical importance^14^.

### Human samples

Human samples analyzed in this study are summarized in Supplementary Table S1 online. The samples were obtained from unrelated individuals. First, we analyzed postmortem cartilage tissues obtained from the knee joints of 22 adolescent/adults (11 women and 11 men). These samples were purchased from Articular Engineering (Catalog IDs, CDD-H-6000-N-1G-R and CDD-H-6000-N-1G-F; Northbrook, IL, USA). The donors of these samples died of trauma or non-endocrine disorders (Supplementary Table S1 online). The samples were either frozen (n = 6) or stored in the RNAlater reagent (Thermo Fisher Scientific, Waltham, MA, USA) (n = 16).

Second, we examined cartilage tissues and cultured chondrocytes obtained from 26 healthy children (11 girls and 15 boys). These samples were collected at our hospital during surgery for polydactyly. Of these, 14 cartilage tissues from five girls and nine boys were frozen in liquid nitrogen immediately after surgery. The remaining 12 cartilage tissues from six girls and six boys were subjected to cartilage culture. The cartilage tissues were pulverized and cultured in Dulbecco's Modified Eagle's Medium containing 17% fetal bovine serum and antibiotics. We confirmed that the cells had chondrocyte-compatible morphological characteristics^15,16^ and expressed *COL2A1*, a marker of chondrocytes.

### Total RNA and genomic DNA extraction

Total RNA of cartilage tissues was extracted using CRYO-PRESS (Microtec, Funabashi, Japan) and the TRIzol reagent (Thermo Fisher Scientific), and that of cultured cells was obtained using the AllPrep DNA/RNA/miRNA Universal kit or the miRNeasy mini kit (QIAGEN, Hilden, Germany). The extracted RNA samples were reverse-transcribed into cDNA using the High-Capacity cDNA reverse transcription kit (Thermo Fisher Scientific). Genomic DNA samples of cartilage tissues were extracted using the Gentra Puregene kit (QIAGEN).

### Primer information

Primers used in this study are listed in Supplementary Table S3 online.

### Microarray-based transcriptome analysis of X chromosomal genes

Transcriptome analyses were performed for cartilage tissues of adults (two women and two men) and cultured chondrocytes of children (six girls and six boys) using catalog one-color microarrays (SurePrint G3 Human Gene Expression microarray, 8 × 60 k format; Agilent Technologies, Santa Clara, CA, USA). The data were analyzed using GeneSpring software (version 14.9, Agilent Technologies). Data were normalized as follows; (i) the 75th percentile value of logarithmically transformed signal intensities within each sample was specified; and (ii) the 75th percentile value was subtracted from the logarithmically transformed signal intensity of each probe. We focused on X chromosomal genes known to escape XCI. Transcripts with low expression levels and low signal quality were filtered out using Feature Extraction software (Agilent Technologies). Specifically, we excluded probes that were assessed as “not uniform,” “saturated,” or “population outliers” in one or more of the tested samples. We also excluded probes that were “not significant” or “not above background” in one or more samples of both sex groups. The female-male ratio at each transcript level was calculated by subtracting the average value of logarithmically transformed signal intensities in the female samples from that in the male samples. Since *SHOX* expression levels were low in some samples, we analyzed its data manually.

### RT-qPCR analysis of *SHOX*

*SHOX* expression levels in the cartilage tissues of 22 adolescent/adults (11 women and 11 men) and 14 children (five girls and nine boys) were analyzed by RT-qPCR. The transcript corresponding to exons 5 and 6a of *SHOX* (assay ID, Hs00757861_m1; Thermo Fisher Scientific) was quantified using TaqMan assays on a 7500 Fast Real-Time PCR system (Thermo Fisher Scientific). *SHOX* expression levels were calculated using the ΔΔ-Ct method against two internal control genes, *TBP* (assay ID, Hs00427620_m1) and *GUSB* (assay ID, Hs00939627_m1). Each sample was analyzed in triplicate.

### RRBS of X chromosomal genes

We performed RRBS of cartilage tissues obtained from four adults (two women and two men), to examine sex differences in the DNA methylation profile of X chromosomal genes. RRBS libraries were prepared according to the standard methods with some modifications^17,18^. Our original protocol started with the sonication of genomic DNA samples, as we found that this process increases the yield after bead purification. The sonication was performed using a Covaris Focused-ultrasonicator S220 (Covaris, Woburn, MA, USA) with a peak incident power of 140 W, a duty cycle of 2%, and a duration of 1 s. The sonicated DNA samples were treated with proteinase K (Thermo Fisher Scientific) and RNase A (Nacalai Tesque, Kyoto, Japan). Subsequently, the samples were bead-purified, digested with MspI (New England BioLabs, Ipswich, MA, USA), subjected to gap filling and A-tailing with a Klenow fragment (Thermo Fisher Scientific), and ligated with the NEBNext methylated adaptor (New England BioLabs). The adaptor-ligated DNA samples were subjected to bisulfite conversion using the EZ DNA Methylation-Gold kit (Zymo Research, Irvine, CA, USA) and amplified by PCR with the KAPA HiFi HotStart Uracil + ReadyMix kit (Roche, Basel, Switzerland). The libraries were subjected to 150-bp paired-end sequencing on a NextSeq sequencer (Illumina, San Diego, CA, USA).

Sequence reads were mapped to the human reference genome for bisulfite sequencing (CT-converted hs37d5) using the Bismark program^19^. Adaptor trimming and quality control were performed using Trim Galore software (https://www.bioinformatics.babraham.ac.uk/projects/trim_galore). The methylation index of each CpG site was calculated using the methylKit package (v.1.10.0) in R^20^. We excluded CpG sites whose read depth was < 10 in any of the tested samples. We also excluded constantly hypermethylated CpG sites with methylation indexes of > 80% in all samples, because this study focused on differentially methylated CpG sites that are possibly involved in epigenetic gene regulation. Sex differences in DNA methylation statuses were calculated by subtracting the mean methylation index of the male samples from that of the female samples.

### Pyrosequencing of *SHOX*-flanking CpG sites

To validate the results of RRBS, we performed pyrosequencing of the *SHOX*-flanking CpG sites using knee cartilage tissues from 22 adolescent/adults (11 women and 11 men). Genomic DNA samples were treated with bisulfite using the EZ DNA Methylation-Gold kit (Zymo Research). Three genomic intervals encompassing CpG clusters (chrX:580,908–581,116, 593,482–593,694, and 605,642–605,771) were PCR-amplified and subjected to pyrosequencing on PyroMark Q24 (QIAGEN). We analyzed methylation indexes of 16 CpG sites (seven in the upstream region, five in intron 2, and four in intron 5–exon 6a).

### Statistical analyses

We conducted Wilcoxon rank sum tests for the results of RT-qPCR. *P* values less than 0.05 were considered to be statistically significant. To evaluate the correlations between the average DNA methylation indexes in the three *SHOX*-flanking regions and *SHOX* expression levels, we calculated the Pearson correlation coefficients and *P* values. Samples in which *SHOX* expression was not detected by RT-qPCR were excluded from statistical analysis. We performed two-tailed Student’s t-tests to examine sex differences in the DNA methylation indexes of the 16 CpG sites in the *SHOX*-flanking regions. Considering that our pyrosequencing analysis targeted 16 CpG sites, we applied the Bonferroni correction for multiple comparisons. *P* values less than 0.0031 (0.05 divided by 16) were considered to be statistically significant.

### Supplementary Information

Supplementary Information.

## Data Availability

All raw and processed microarray and sequencing data generated in this study are available at the NCBI Gene Expression Omnibus with the accession number GSE188531. The data will be open to the public after the manuscript is accepted.
