# Supplementary material for: Expression levels and DNA methylation profiles of the growth gene SHOX in cartilage tissues and chondrocytes
Source: Sci Rep. 2024 Apr 5;14:8069. doi: 10.1038/s41598-024-58530-9 (PMC10997625; doi:10.1038/s41598-024-58530-9)
Supplement: Supplementary file 1 — Supplementary Information. [file 41598_2024_58530_MOESM1_ESM.docx]

**Supplementary information**

1. **Supplementary Figure S1**
2. **Supplementary Tables S1–S3**
3. **References for Supplementary information**

**Supplementary Figure**


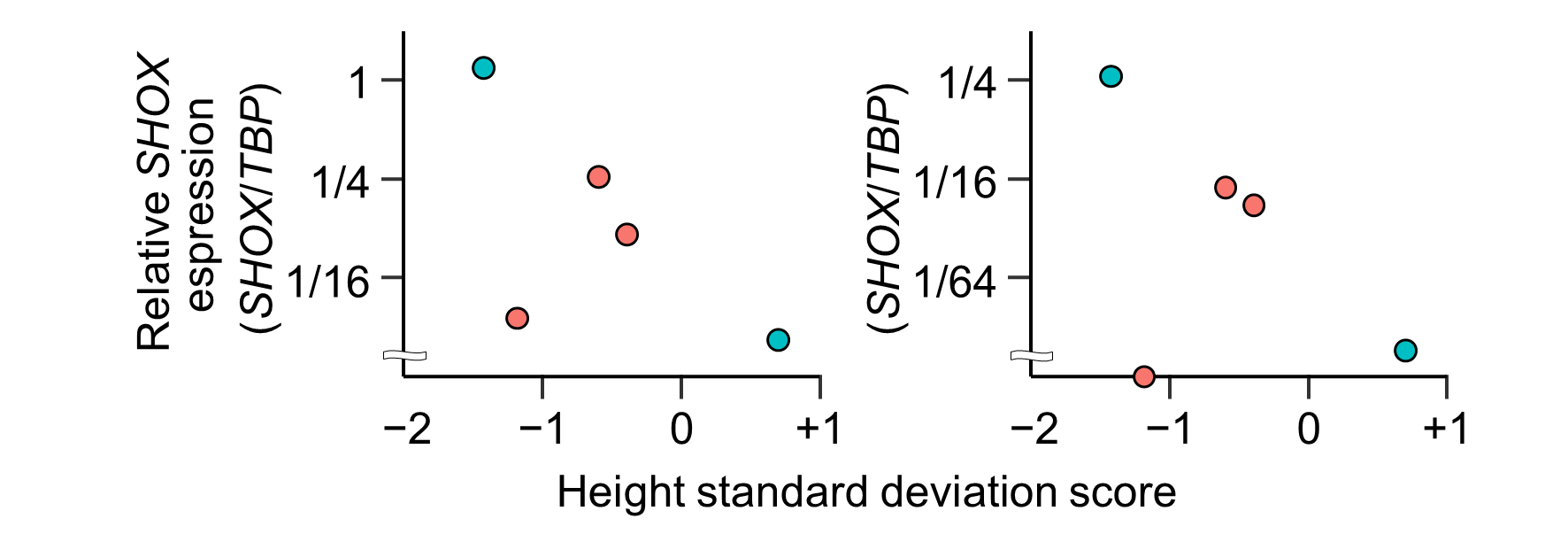


# **Supplementary Figure S1. Correlation between *SHOX* expression levels and height in five children.**

The X-axis represents the height standard deviation scores of the patients at the latest visit. The Y-axis represents the relative *SHOX* expression levels against *TBP* or *GUSB*. Blue and red dots represent the data of boys and girls, respectively. Height data of other participants were unavailable.

# **SUPPLEmentary Tables**

# **Supplementary Table S1. Human samples analyzed in this study.**

|  | Sample | |  | Donor information | | |  | Experiments | | | |
| --- | --- | --- | --- | --- | --- | --- | --- | --- | --- | --- | --- |
|  | Sample ID | Sample type |  | Age | Sex | Disorder |  | Transcriptome analysis | RT-qPCR | RRBS | Pyro-sequencing |
| Cartilage tissues obtained from postmortem knee samples | | | | | | | | | | | |
|  | ACa01^a^ | RNAlater^c^ |  | 45 y | F | Anoxic brain injury |  | ✓ | ✓ | ✓ | ✓ |
|  | ACa02^a^ | RNAlater^c^ |  | 55 y | F | Nasal cancer |  | ✓ | ✓ | ✓ | ✓ |
|  | ACa03^b^ | Fresh frozen |  | 58 y | F | Cerebrovascular accident |  |  | ✓ |  | ✓ |
|  | ACa04^b^ | Fresh frozen |  | 58 y | F | Cardiopulmonary arrest |  |  | ✓ |  | ✓ |
|  | ACa05^b^ | Fresh frozen |  | 55 y | F | Trauma |  |  | ✓ |  | ✓ |
|  | ACa06^b^ | Fresh frozen |  | 54 y | F | Myocardial infection |  |  | ✓ |  | ✓ |
|  | ACa07^a^ | RNAlater^c^ |  | 55 y | M | Lung cancer |  | ✓ | ✓ | ✓ | ✓ |
|  | ACa08^a^ | RNAlater^c^ |  | 54 y | M | Head trauma |  | ✓ | ✓ | ✓ | ✓ |
|  | ACa09^b^ | Fresh frozen |  | 52 y | M | Pulmonary embolism |  |  | ✓ |  | ✓ |
|  | ACa10^b^ | Fresh frozen |  | 53 y | M | Trauma |  |  | ✓ |  | ✓ |
|  | ACa11^b^ | RNAlater^c^ |  | 56 y | M | Squamous cell carcinoma |  |  | ✓ |  | ✓ |
|  | ACa12^b^ | RNAlater^c^ |  | 74 y | M | Congestive heart failure |  |  | ✓ |  | ✓ |
|  | ACa13^b^ | RNAlater^c^ |  | 64 y | M | Pancreatic cancer |  |  | ✓ |  | ✓ |
|  | ACa14^b^ | RNAlater^c^ |  | 65 y | F | Lung cancer |  |  | ✓ |  | ✓ |
|  | ACa15^b^ | RNAlater^c^ |  | 77 y | M | Cardiopulmonary failure |  |  | ✓ |  | ✓ |
|  | ACa16^b^ | RNAlater^c^ |  | 14 y | M | Small cell osteosarcoma |  |  | ✓ |  | ✓ |
|  | ACa17^b^ | RNAlater^c^ |  | 48 y | F | Multiple sclerosis |  |  | ✓ |  | ✓ |
|  | ACa18^b^ | RNAlater^c^ |  | 39 y | M | Conseequences of anoxic brain injury |  |  | ✓ |  | ✓ |
|  | ACa19^b^ | RNAlater^c^ |  | 43 y | F | Cirrhosis |  |  | ✓ |  | ✓ |
|  | ACa20^b^ | RNAlater^c^ |  | 63 y | F | Lung cancer |  |  | ✓ |  | ✓ |
|  | ACa21^b^ | RNAlater^c^ |  | 73 y | F | Chronic obstructive pulmonary disease |  |  | ✓ |  | ✓ |
|  | ACa22^b^ | RNAlater^c^ |  | 67 y | M | Alzheimer’s disease |  |  | ✓ |  | ✓ |
| Chondrocyte cell lines established from surgical specimens of children with polydactyly | | | | | | | | | | | |
|  | CCh01 | Cultured cells |  | 11 m | F | Polydactyly |  | ✓ |  |  |  |
|  | CCh02 | Cultured cells |  | 17 m | F | Polydactyly |  | ✓ |  |  |  |
|  | CCh03 | Cultured cells |  | 13 m | F | Polydactyly |  | ✓ |  |  |  |
|  | CCh04 | Cultured cells |  | 12 m | F | Polydactyly |  | ✓ |  |  |  |
|  | CCh05 | Cultured cells |  | 13 m | F | Polydactyly |  | ✓ |  |  |  |
|  | CCh06 | Cultured cells |  | 17 m | F | Polydactyly |  | ✓ |  |  |  |
|  | CCh07 | Cultured cells |  | 15 m | M | Polydactyly |  | ✓ |  |  |  |
|  | CCh08 | Cultured cells |  | 12 m | M | Polydactyly |  | ✓ |  |  |  |
|  | CCh09 | Cultured cells |  | 16 m | M | Polydactyly |  | ✓ |  |  |  |
|  | CCh10 | Cultured cells |  | 17 m | M | Polydactyly |  | ✓ |  |  |  |
|  | CCh11 | Cultured cells |  | 13 m | M | Polydactyly |  | ✓ |  |  |  |
|  | CCh12 | Cultured cells |  | 19 m | M | Polydactyly |  | ✓ |  |  |  |
| Cartilage tissues obtained from children with polydactyly | | | | | | | | | | | |
|  | CCa01 | Fresh frozen |  | 11 m | F | Polydactyly |  |  | ✓ |  |  |
|  | CCa02 | Fresh frozen |  | 14 m | F | Polydactyly |  |  | ✓ |  |  |
|  | CCa03 | Fresh frozen |  | 16 m | F | Polydactyly |  |  | ✓ |  |  |
|  | CCa04 | Fresh frozen |  | 13 m | F | Polydactyly |  |  | ✓ |  |  |
|  | CCa05 | Fresh frozen |  | 12 m | F | Polydactyly |  |  | ✓ |  |  |
|  | CCa06 | Fresh frozen |  | 13 m | M | Polydactyly |  |  | ✓ |  |  |
|  | CCa07 | Fresh frozen |  | 10 m | M | Polydactyly |  |  | ✓ |  |  |
|  | CCa08 | Fresh frozen |  | 12 m | M | Polydactyly |  |  | ✓ |  |  |
|  | CCa09 | Fresh frozen |  | 12 m | M | Polydactyly |  |  | ✓ |  |  |
|  | CCa10 | Fresh frozen |  | 14 m | M | Polydactyly |  |  | ✓ |  |  |
|  | CCa11 | Fresh frozen |  | 12 m | M | Polydactyly |  |  | ✓ |  |  |
|  | CCa12 | Fresh frozen |  | 9 m | M | Polydactyly |  |  | ✓ |  |  |
|  | CCa13 | Fresh frozen |  | 19 m | M | Polydactyly |  |  | ✓ |  |  |
|  | CCa14 | Fresh frozen |  | 11 m | M | Polydactyly |  |  | ✓ |  |  |

F, female; M, male; RRBS, reduced representation bisulfite sequencing; m, month; y, year.

^a^Catalog ID, CDD-H-6000-N-1G-R

^b^Catalog ID, CDD-H-6000-N-1G-F

^c^Treated with the RNAlater reagent (Thermo Fisher Scientific, MA, USA)

# **Supplementary Table S2. Previously reported male individuals with *SHOX* abnormalities on the X and Y chromosomes.**

|  | Case | Age (years) | Height (SD) | Madelung deformity | *SHOX* variant | Reference |
| --- | --- | --- | --- | --- | --- | --- |
| Individuals with *SHOX* abnormalities on the X chromosome | | | | | | |
|  | Patient 34 | 8.9 | −3.06 | − | duplication involving CNE−3 | [1] |
|  | Patient 5 | 7.5 | −2.7 | − | c.503G>A (p.Arg168Gln) | [2] |
|  | Patient | 5.9 | −1.99 | + | c.353G>T (p.Arg118Met) | [3] |
|  | Patient 2 | 12.3 | −3.1 | − | deletion involving CNE−3–ECS4/CNE9 | [4] |
|  | Patient 4 | 10.5 | −2.7 | − | c.698C>T (p.Ala233Val) | [4] |
|  | Patient 6 | 8.5 | −2.6 | − | deletion involving ECS4/CNE9 | [4] |
|  | 91-Proband | adult | −2.3 | + | deletion involving CNE7 | [5] |
|  | 141-Proband | 12 | −3 | − | deletion involving CNE7 | [5] |
|  | 10-II:1 | no data | −2.47 | + | deletion involving CNE7 | [6] |
|  | 12-II:1 | no data | −3.02 | + | deletion involving CNE7 | [6] |
|  | 15-II:2 | no data | −2.14 | + | deletion involving CNE7 | [6] |
|  | 26-II:1 | no data | −2.56 | − | deletion involving CNE7 | [6] |
|  | 4-II:1 | 3.5 | −2.7 | + | c.509C>A (p.Ala170Pro) | [7] |
|  | 5-II:1 | 14.4 | −2.4 | + | c.509C>A (p.Ala170Pro) | [7] |
|  | 11-II:2 | 10 | −2.06 | + | deletion involving exons 2–6a | [8] |
|  | IX-II:3 | adult | −2.6 | no data | c.105C>A (p.Tyr35*) | [9] |
|  | IX-II:6 | adult | −2.9 | no data | c.105C>A (p.Tyr35*) | [9] |
|  | IX-II:13 | adult | −2.4 | no data | c.105C>A (p.Tyr35*) | [9] |
|  | Case 38 | no data | −1.8 | − | downstream deletion | [10] |
|  | Case 42 | no data | −3.0 | − | c.178A>C (p.Thr60Pro) | [10] |
|  | Patient 5 | 15.9 | −1.77 | + | c.355C>G (p.Arg119Gly) | [11] |
|  | Patient 16 | 17.1 | −2.85 | + | c.877T>C (p.*293Arg) | [11] |
|  | A2 | 6.4 | −2.6 | − | c.583C>T (p.Arg195*) | [12] |
| Individuals with *SHOX* abnormalities on the Y chromosome | | | | | | |
|  | Patient | 7.9 | −3.0 | − | downstream deletion | [13] |
|  | Patient #2 | 15.8 | −2.3 | + | downstream deletion | [14] |
|  | Patient 17 | 4.5 | −0.98 | − | deletion involving CNE7 | [1] |
|  | Patient 18 | 5.6 | −1.53 | − | deletion involving CNE7 | [1] |
|  | 117-Proband | adult | −2.3 | + | deletion involving CNE7 | [5] |
|  | 16-II:1 | no data | −2.52 | − | deletion involving CNE7 | [6] |
|  | 17-II:1 | no data | −0.18 | + | deletion involving CNE7 | [6] |
|  | 25-II:1 | no data | −2.79 | − | deletion involving CNE7 | [6] |
|  | Patient 1 | 13.2 | −2.02 | − | downstream deletion | [15] |
|  | Patient 5 | 10.7 | −3.28 | − | c.685_696del (p.His229_Leu232del) | [15] |
|  | P3 | no data | −2.0 | − | deletion involving ECS4/CNE9 | [16] |
|  | 11-III:8 | adult | −4.1 | + | c.509C>A (p.Ala170Pro) | [7] |
|  | 7-II:1 | 10.7 | −1.80 | + | deletion involving exons 2–6b | [8] |
|  | IX-III:6 | 11.8 | −2 | − | c.105C>A (p.Tyr35*) | [9] |
|  | IX-III:7 | no data | −2.4 | no data | c.105C>A (p.Tyr35*) | [9] |
|  | Case 12 | no data | −1.8 | + | c.463G>C (p.Gly155Arg) | [10] |
|  | Case 40 | no data | −1.8 | − | c.643C>T (p.Gln215*) | [10] |
|  | Patient 20 | 6.1 | −2.61 | + | c.304G>T (p.Glu102*) | [11] |

CNE, evolutionarily conserved non-coding DNA element; ECS, evolutionarily conserved sequence; SD, standard deviation.

# **Supplementary Table S3. Primers used in this study.**

|  |  | Forward | Reverse | Sequencing |
| --- | --- | --- | --- | --- |
| Amplification of reduced representation bisulfite sequencing (RRBS) libraries | | | | |
|  | Universal primer (P5) | AATGATACGGCGACCACCGA |  |  |
|  | Universal primer (P7) | CAAGCAGAAGACGGCATACGA |  |  |
| Pyrosequencing | | | | |
|  | *SHOX* upstream | AGGAAGAGGTATATTTTGTTTTTTAAGATT | ACCTAATCCCCCCAAAACTAACCCTA | GGTAGAAAGAGAATGGTATTTAGG |
|  | Intron 2 | GTTAGGGGGAGAAGAGAGAGT | AAATTCCTCCCTACACACTACT | AATTAAAAAGTAATTTTATAGAAG |
|  | Exon 6a-1 | GGGATTTTGGAGAAGGGTAAATT | AACCAACTCCCAAATCTTTACAATTTCAA | TGGAGAAGGGTAAATTT |
|  | Exon 6a-2 | GGGATTTTGGAGAAGGGTAAATT | AACCAACTCCCAAATCTTTACAATTTCAA | GTTTTTTTTTGTTATATTTTATGTA |

P5 and P7 primers comprise sequences that bind to the capture nucleotides of the Illumina platform.

# **References for Supplementary information**

1. Capkova, P., Capkova, Z., Rohon, P., Adamová, K. & Zapletalova, J. Short stature and SHOX (Short stature homeobox) variants—efficacy of screening using various strategies. *PeerJ* **8**, e10236 (2020).

2. Freire, B. L. *et al.* Multigene sequencing analysis of children born small for gestational age with isolated short stature. *J Clin Endocrinol Metab* **104**, 2023–2030 (2019).

3. Lucchetti, L. *et al.* Report of a novel SHOX missense variant in a boy with short stature and his mother with Leri–Weill dyschondrosteosis. *Front. Endocrinol.* **9**, 163 (2018).

4. Tung, Y.-C. *et al.* SHOX deficiency in short Taiwanese children: a single-center experience. *J. Formos. Med. Assoc.* **117**, 909–914 (2018).

5. Bunyan, D. J., Baker, K. R., Harvey, J. F. & Thomas, N. S. Diagnostic screening identifies a wide range of mutations involving the SHOX gene, including a common 47.5 kb deletion 160 kb downstream with a variable phenotypic effect. *Am. J. Med. Genet. A* **161A**, 1329–1338 (2013).

6. Benito-Sanz, S. *et al.* Identification of the first recurrent PAR1 deletion in Léri-Weill dyschondrosteosis and idiopathic short stature reveals the presence of a novel SHOX enhancer. *J. Med. Genet.* **49**, 442–450 (2012).

7. Barca-Tierno, V. *et al.* Identification of a Gypsy SHOX mutation (p.A170P) in Léri-Weill dyschondrosteosis and Langer mesomelic dysplasia. *Eur. J. Hum. Genet.* **19**, 1218–1225 (2011).

8. Benito-Sanz, S. *et al.* Clinical and molecular evaluation of SHOX/PAR1 duplications in Léri-Weill dyschondrosteosis (LWD) and idiopathic short stature (ISS). *J. Clin. Endocrinol. Metab.* **96**, E404–E412 (2011).

9. Jorge, A. A. L. *et al.* SHOX mutations in idiopathic short stature and Leri-Weill dyschondrosteosis: frequency and phenotypic variability. *Clin. Endocrinol. (Oxf.)* **66**, 130–135 (2007).

10. Huber, C. High incidence of SHOX anomalies in individuals with short stature. *J. Med. Genet.* **43**, 735–739 (2006).

11. Binder, G. *et al.* SHOX haploinsufficiency and Leri-Weill dyschondrosteosis: prevalence and growth failure in relation to mutation, sex, and degree of wrist deformity. *J. Clin. Endocrinol. Metab.* **89**, 4403–4408 (2004).

12. Rao, E. *et al.* Pseudoautosomal deletions encompassing a novel homeobox gene cause growth failure in idiopathic short stature and Turner syndrome. *Nat. Genet.* **16**, 54–63 (1997).

13. Fukami, M., Shindo, J., Ogata, T., Kageyama, I. & Kamimaki, T. SHOX far-downstream deletion in a patient with nonsyndromic short stature. *Am J Med Genet A* **188**, 2173–2177 (2022).

14. Fanelli, A. *et al.* Copy number variations residing outside the SHOX enhancer region are involved in Short Stature and Léri-Weill dyschondrosteosis. *Mol Genet Genomic Med* **10**, e1793 (2022).

15. Caliebe, J. *et al.* IGF1, IGF1R and SHOX mutation analysis in short children born small for gestational age and short children with normal birth size (idiopathic short stature). *Horm. Res. Paediatr.* **77**, 250–260 (2012).

16. Hirschfeldova, K. *et al.* SHOX gene defects and selected dysmorphic signs in patients of idiopathic short stature and Léri–Weill dyschondrosteosis. *Gene* **491**, 123–127 (2012).
